# TGF-β Activity of a Demineralized Bone Matrix

**DOI:** 10.3390/ijms22020664

**Published:** 2021-01-11

**Authors:** Layla Panahipour, Anes Omerbasic, Jila Nasirzade, Reinhard Gruber

**Affiliations:** 1 Department of Oral Biology, Medical University of Vienna, Sensengasse 2a, 1090 Vienna, Austria; layla.panahipour@meduniwien.ac.at (L.P.); anes.omerbasic@hotmail.com (A.O.); jila.nasirzaderajiri@meduniwien.ac.at (J.N.); 2 Department of Periodontology, School of Dental Medicine, University of Bern, Freiburgstrasse 7, 3010 Bern, Switzerland; 3 Austrian Cluster for Tissue Regeneration, Donaueschingenstraße 13, 1200 Vienna, Austria

**Keywords:** demineralized bone matrix, transforming growth factor β1, allografts, bioassay, bone regeneration, bone augmentation

## Abstract

Allografts consisting of demineralized bone matrix (DBM) are supposed to retain the growth factors of native bone. However, it is not clear if transforming growth factor β1 (TGF-β1) is maintained in the acid-extracted human bone. To this aim, the aqueous solutions of supernatants and acid lysates of OraGRAFT^®^ Demineralized Cortical Particulate and OraGRAFT^®^ Prime were prepared. Exposing fibroblasts to the aqueous solution caused a TGF-β receptor type I kinase-inhibitor SB431542-dependent increase in interleukin 11 (IL11), NADPH oxidase 4 (NOX4), and proteoglycan 4 (PRG4) expression. Interleukin 11 expression and the presence of TGF-β1 in the aqueous solutions were confirmed by immunoassay. Immunofluorescence further confirmed the nuclear translocation of Smad2/3 when fibroblasts were exposed to the aqueous solutions of both allografts. Moreover, allografts released matrix metalloprotease-2 activity and blocking proteases diminished the cellular TGF-β response to the supernatant. These results suggest that TGF-β is preserved upon the processing of OraGRAFT^®^ and released by proteolytic activity into the aqueous solution.

## 1. Introduction

Bone regeneration is an evolutionally conserved process that follows the principles of bone development during growth [1]. Bone regeneration is not only required to bridge large defects; it is also a common principle of regenerative medicine extending towards the osseointegration of dental implants [2]. However, bone atrophy occurring as a consequence of tooth loss [3] or periodontal and peri-implant inflammatory osteolysis [4] necessitates bone reconstruction before or simultaneously to dental implant placement. Bone reconstruction or bone augmentation requires the lost bone to be replaced; this can be achieved by autografts [5], allografts [6], synthetic biomaterials [7], deproteinized bone of xenogeneic origin [8], and marine-derived materials [9] applied to dentistry and craniofacial surgery.

Autografts and allografts are unique in that they either match the bone at the defect site or are processed to maintain at least part of the original properties, respectively [10,11]. Even though autografts are considered ideal to support bone regeneration considering their favorable osteoconductive and osteogenic properties, the amount is limited and harvesting is invasive [12,13]. Allografts provide a viable alternative to autografts as they are produced from human bone that usually undergoes defatting, demineralization, virus inactivation, and gamma sterilization [14]. Each provider of allografts has established their own protocol to produce allografts, particularly demineralized bone matrix (DBM). Even though the basic protocols are similar, they are not the same and consequently the biological, and presumably, also the clinical, properties of the various DBMs might differ from each other. The question arising is how the processing of a donor’s bone affects the biological properties of the DBM allograft.

Research on allografts dates back to the time when the osteoinductive properties of DBM were reported in rodent and rabbit models [15,16,17]. The osteoinductive properties of DBM are caused by the bone morphogenetic proteins, which all belong to the transforming growth factor β (TGF-β) superfamily of growth factors. Apart from the rather low amount of bone morphogenetic proteins (BMPs) in bone [18,19,20], bone is a rich source of TGF-β [21,22,23], a multifunctional growth factor playing a major role in bone regeneration and remodeling [24,25]. It might be not surprising that immunoassays could detect TGF-β1 released from mineralized allografts [26]. However, the situation for DBM is less clear. Significant amounts of TGF-β1 were extractable only from one out of the three commercially available products containing DBM [27]. Moreover, immunoassays do not necessarily indicate the biological activity of the released TGF-β1. Consequently, there is a demand to identify the TGF-β1 activity released by DBM.

The binding of a released TGF-β to the respective receptor dimer causes the activation of the TGF-β receptor type I kinase-inhibitor that phosphorylates the cytoplasmic Smad2 and Smad3 messengers that, upon dimerization, translocate into the nucleus and change the transcript of the TGF-β target genes [25,28]. Among those TGF-β target genes are interleukin 11 (IL11), NADPH oxidase 4 (NOX4), and proteoglycan 4 (PRG4), which are identified by screening approaches when mesenchymal cells are exposed to acid bone lysates [23], acid dentine lysates (unpublished), bone-conditioned medium [29], and enamel matrix derivatives [30]. Support for selecting those genes to identify the TGF-β activity comes from blocking the TGF-β receptor type I kinase with the inhibitor SB431542 [23,29,30]. This canonical, biological cell response can be identified at the molecular level and can serve as a bioassay to identify TGF-β1 activity released by DBM.

The clinical implication of identifying TGF-β1 activity released by DBM remains vague. Nevertheless, showing that TGF-β activity is released from DBM is an indication that the processing of the original bone into an allograft maintains part of the original growth factor activity. Measuring TGF-β1 activity might become useful in monitoring the manufacturing process of DBM. Our research also paves the way for future preclinical research with the aim to determine to which extent the TGF-β activity of DBM supports graft consolidation. Here, we took advantage of the bioassay approach to identify TGF-β activity being released from OraGRAFT^®^ Demineralized Cortical Particulate and OraGRAFT^®^ Prime.

## 2. Results

### 2.1. Cell Viability in Response to Supernatants and Lysates of OraGRAFT^®^

To screen for a possible impact of cell viability, gingival fibroblasts were exposed to various dilutions of the aqueous solutions of OraGRAFT^®^ Demineralized Cortical Particulate (SC) and OraGRAFT^®^ Prime consisting of moldable fibers (SF). At no concentration tested did the supernatant (SC, SF) negatively affect cell viability. Acid lysates of OraGRAFT^®^ Demineralized Cortical Particulate (LC) and acid lysates of OraGRAFT^®^ Prime (LF) showed a different response (Table 1). We observed that the treatment of cells with more than 12% and 25% of LC and LF, respectively, decreased cell viability. As a consequence, for the bioassays, cells were treated with 30% of the supernatant (SC, SF), 6% LC, and 12% LF.

### 2.2. TGF-β1 Identified by Immunoassay in Supernatants and Lysates of OraGRAFT^®^

**Figure 1:**
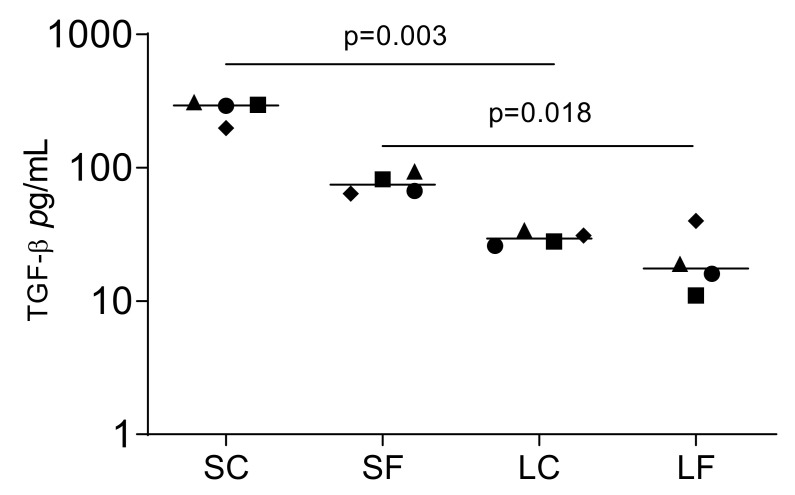
Supernatants that are the aqueous solution of OraGRAFT^®^ Demineralized Cortical Particulate (SC) and OraGRAFT^®^ Prime (SF) contain TGF-β1. The immunoassay identified less TGF-β1 in the acid lysates of OraGRAFT^®^ (LC and LF). N = 4. Statistic is based on a paired *t*-test.

Proteomics can identify TGF-β1 in the acid lysates of porcine bone [23]. To determine if OraGRAFT^®^ can release TGF-β1 spontaneously into an aqueous solution, we measured the concentration of TGF-β1 by immunoassay. Undiluted SC and SF contain 294.0 pg/mL TGF-β1 and 74.5 pg/mL TGF-β1, respectively (Figure 1). This represents approximately 1.4 ng TGF-β1 and 0.6 ng TGF-β1 released per unit of OraGRAFT^®^ Demineralized Cortical Particulate and OraGRAFT^®^ Prime, respectively. TGF-β1 was lower in undiluted LC and LF, which was in median 29.5 pg/mL and 17.5 pg/mL TGF-β, respectively.

### 2.3. TGF-β1 Activity by Bioassay in Supernatants and Lysates of OraGRAFT^®^

**Figure 2:**
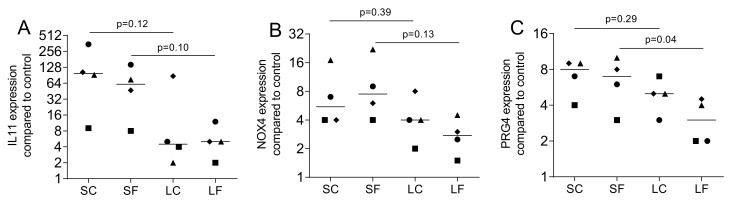
Gene expression of fibroblasts exposed to the supernatants and acid lysates of allografts. Gingival fibroblasts were exposed to aqueous supernatants of OraGRAFT^®^ Demineralized Cortical Particulate (SC) and OraGRAFT^®^ Prime (SF) and the acid lysates of the two respective allografts (LC and LF). The expression of (**A**) interleukin 11 (IL11), (**B**) NADPH oxidase 4 (NOX4), and (**C**) proteoglycan 4 (PRG4) was measured by quantitative RT-PCR. N = 4. Statistic is a paired *t*-test.

We next evaluated if the TGF-β1 released from OraGRAFT has a biological activity. Gingival fibroblasts were exposed to the supernatant (SC and SF) and acid lysates (LC and LF) of OraGRAFT^®^. Gene expression analysis revealed a robust increase of IL11, NOX4, and PRG4 (Figure 2). The supernatants of OraGRAFT^®^ (SC and SF) were more potent to increase gene expression than the respective acid lysates (LC and LF).

**Figure 3:**
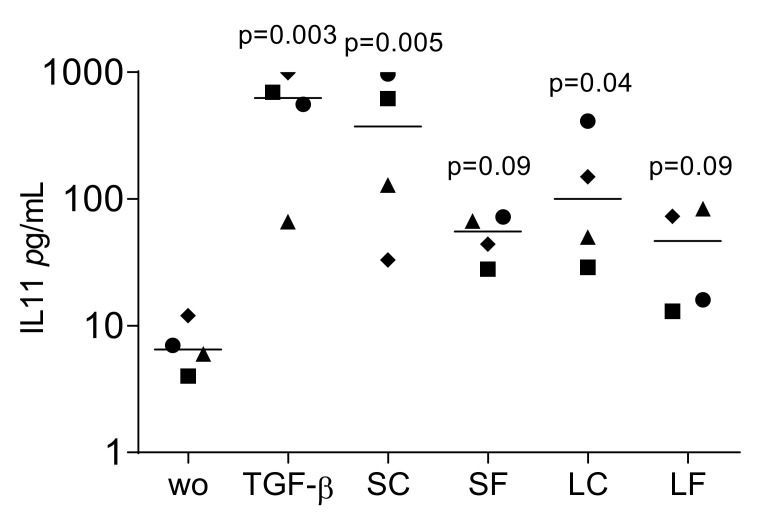
IL11 levels in the culture medium of fibroblasts exposed to supernatants and acid lysates of allografts. Gingival fibroblasts were exposed to aqueous supernatants of OraGRAFT^®^ Demineralized Cortical Particulate (SC) and OraGRAFT^®^ Prime (SF) and the acid lysates of the two respective allografts (LC and LF). The IL11 levels were measured by immunoassay. N = 4. Statistic is an uncorrected Friedmann test against untreated controls.

To confirm the translation of gene expression into protein signals, IL11 levels in the culture medium of fibroblasts were evaluated. The supernatants from OraGRAFT^®^ Demineralized Ground Cortical (SC) and its respective acid lysate (LC) significantly increased the release of IL11 by the gingival fibroblasts. (Figure 3). Supernatants of OraGRAFT^®^ Prime (SF) and its respective acid lysate were less potent, but also stimulated the production of IL11.

### 2.4. Blocking the TGF-β Receptor Type I Kinase Activity by SB431542

**Figure 4:**
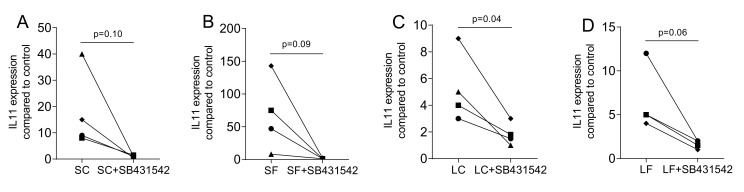
TGF-β receptor type I kinase with the inhibitor SB431542. Gingival fibroblasts were exposed to aqueous supernatants of (**A**) OraGRAFT^®^ Demineralized Cortical Particulate (SC) and (**B**) OraGRAFT^®^ Prime (SF) and (**C**,**D**) the acid lysates of the two respective allografts (LC and LF). The expression of IL11 was diminished by SB431542, the TGF-β receptor type I kinase. N = 4. Statistic is a paired *t*-test.

To confirm that the changes in gene expression are mediated via the TGF-β receptor type I kinase signaling, the antagonist SB431542 was used. As indicated in Figure 4, SB431542 greatly abolished the expression of IL11 that was provoked by exposing the gingival fibroblasts to supernatants of OraGRAFT^®^ Demineralized Cortical Particulate (SC) and OraGRAFT^®^ Prime (SF) as well as to the acid lysates (LC and LF).

### 2.5. TGF-β1 Activity by Immunoassay in Supernatants of OraGRAFT^®^

**Figure 5:**
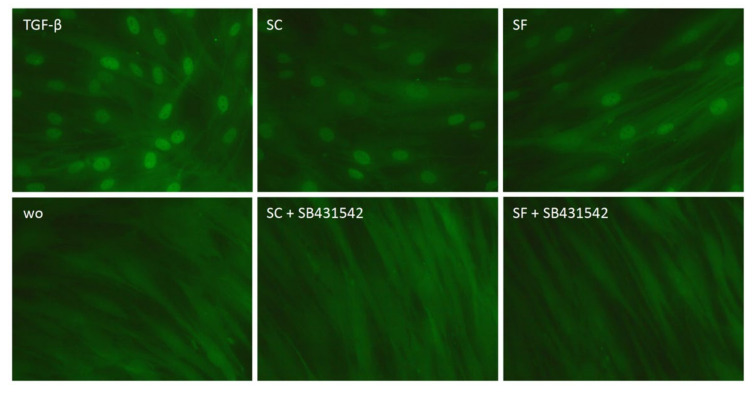
Nuclear translocation of Smad2/3 in response to the allograft supernatants. Gingival fibroblasts were exposed to aqueous supernatants of OraGRAFT^®^ Demineralized Cortical Particulate (SC) and OraGRAFT^®^ Prime (SF) with and without the TGF-β receptor type I kinase-inhibitor SB431542. Recombinant TGF-β1 served as a positive control. Without (wo) is the serum-free medium alone. The positive signals induced by SC and SF are indicated by the nuclear staining that is blocked by the SB431542.

To underpin the TGF-β activity, gingival fibroblasts were exposed to the supernatant of OraGRAFT^®^ and the translocation of Smad2/3 was analyzed by immunostaining (Figure 5). We observed that the supernatant of OraGRAFT^®^ Demineralized Cortical Particulate (SC) and OraGRAFT^®^ Prime (SF), both and similar to recombinant TGF-β1, caused a nuclear translocation of Smad2/3 but not in the presence of the TGF-β receptor type I kinase-inhibitor SB431542.

### 2.6. Gelatin Zymography of Supernatants and Lysates of OraGRAFT^®^

**Figure 6:**
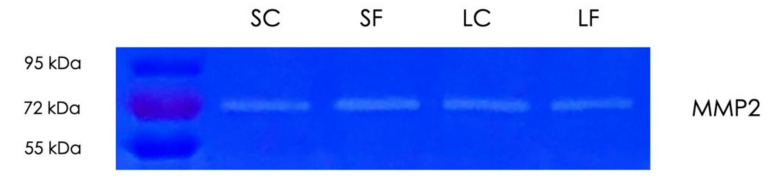
Gelatin zymography of allograft supernatants and acid lysates. Aqueous supernatants of OraGRAFT^®^ Demineralized Cortical Particulate (SC) and OraGRAFT^®^ Prime (SF) and the respective acid lysates (LC and LF) were subjected to gelatin zymography. The bands representing the digestion of gelatin indicate the presence of matrix metalloproteinase-2 (MMP2).

Considering that TGF-β, which is adsorbed by decorin linked to collagen, is released by matrix metalloproteinases (MMPs) [31] and MMPs are involved in TGF-β activation [32], we have tested the gelatinolytic activity of the supernatants and the acid lysates of OraGRAFT^®^ Demineralized Cortical Particulate (SC and LC) and OraGRAFT^®^ Prime (SF and LF) by zymography (Figure 6). We noticed a single band that indicates the presence of MMP2, but not MMP9, in all preparations. Thus, the supernatants and the acid lysates of the allografts digest gelatin.

### 2.7. Blocking of Protease Activity Reduces TGF-β1 Activity in Supernatants of OraGRAFT^®^

**Figure 7:**
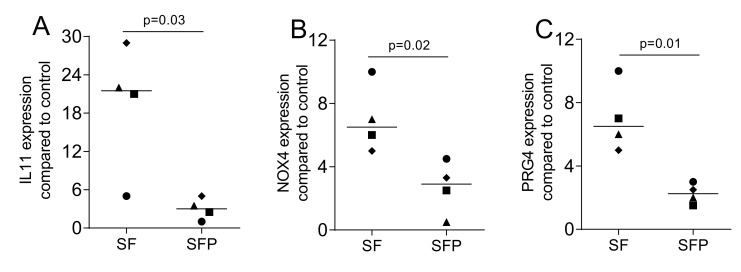
Protease inhibitors lower the TGF-β activity in the supernatant of OraGRAFT^®^ Prime. Gingival fibroblasts were exposed to aqueous supernatants of OraGRAFT^®^ Prime in the absence (SF) or presence (SFP) of the protease inhibitors. The expression of (**A**) IL11, (**B**) NOX4, and (**C**) PRG4 was decreased significantly by protease inhibitors. The expression of IL11, NOX4, and PRG4 was measured by quantitative RT-PCR. N = 4. Statistic is a paired *t*-test.

Finally, we blocked proteases and measured the TGF-β1 activity released from the allograft. (Figure 7). The presence of protease inhibitors reduced TGF-β1 activity of the aqueous supernatants of OraGRAFT^®^ Prime, as indicated by the expression of IL11, NOX4, and PRG4, compared with preparations without the inhibitors. Accordingly, the proteases intrinsic to allografts are involved in the release and/or the activation of TGF-β1.

## 3. Discussion

Allograft DBM is widely applied in regenerative medicine, particularly to reconstruct bone defects that otherwise would not heal adequately. In dentistry, the reconstruction of bone that is lost due to atrophy [3] or as a consequence of inflammatory osteolysis [4] is a prerequisite for the placement of dental implants. It was particularly the osteoinductive property of DBM that have inspired the search for growth factors [15,16,17], ultimately leading to the discovery of the members of the BMP family [18,19,20]. Today, recombinant BMP2 is clinically approved for bone graft in sinus augmentation and localized alveolar ridge augmentation [33,34], as well as spinal fusion procedures in skeletally mature patients with degenerative disc disease [35]. TGF-β, despite giving the name for the superfamily, has not been studied extensively in the context of DBM [27]. This is likely because one might assume that TGF-β, which is rich in the original bone matrix, is removed by the acid lysis of the demineralization step. In contrast to this assumption, we show here that the aqueous solution of OraGRAFT^®^ allografts not only releases TGF-β1 detected by immunoassay, but that the aqueous solution has the capacity to increase IL11, NOX4, and PRG4 expression in gingival fibroblasts. Our data further suggest that gene expression changes require the activation of the TGF-β receptor type I kinase as well as the activation of the Smad2/3 downstream signaling pathway. Finally, we show that the release of TGF-β activity from the OraGRAFT^®^ allografts requires the intrinsic proteases.

If we relate the findings to those of others, we can confirm previous observations where immunoassays have identified TGF-β1 in guanidine HCl-treated DBM of one manufacturer, whereas two other products have negligible amounts of TGF-β1 [27]. Thus, DBM is not necessarily a synonym for acid-soluble TGF-β. Moreover, TGF-β detected by immunoassay upon guanidine HCl treatment does not indicate TGF-β activity. Here, we used an aqueous extraction to release a TGF-β activity from OraGRAFT^®^ products. Our findings suggest that processing of the original allogenic bone into a final product allows for TGF-β activity to be preserved; processing means defatting and clearing, virus inactivation and sterilization, and a process termed the Allowash XG [36]. Moreover, processing of the original allogenic bone maintains at least part of the original MMP2 activity, a protease known to activate latent TGF-β [37]. Our data support the notion that the proteolytic activity of the allografts contributes to the liberation and activation of the TGF-β of the extracellular matrix. Taken together, our observations extend existing knowledge on allograft TGF-β activity.

The clinical relevance remains a matter of debate, but the data suggest that the OraGRAFT^®^ products release a TGF-β activity that becomes available to the cells at the augmentation site. If the host cells indeed respond to the TGF-β, considering that TGF-β1 attracts mesenchymal cells capable of becoming bone-forming osteoblasts during remodeling [24], then OraGRAFT^®^’s TGF-β might affect the overall process of graft consolidation. However, this is a hypothesis, rather than a conclusion, which leaves room for future research. We can further assume that the TGF-β activity of DBM is an easy-to-handle predictor, a surrogate parameter of a BMP activity that is usually tested by ectopic implantation in a preclinical setting [15,16,17,38]. Both molecules are rather similar in nature, being resistant to the low pH required for demineralization and heating becoming important in virus inactivation [18,19,20,39]. The bioassay presented here can be further standardized towards a reporter cell line that allows screening the TGF-β activity for quality control while manufacturing the DBM [40], considering that testing the osteoinductive activity of each batch is not feasible on an industrial level.

The study presented here leaves many questions unanswered. For instance, by which mechanism is TGF-β released into the aqueous fraction and why is the amount of TGF-β lower in the respective acid lysates of the OraGRAFT^®^ product? Our observations that the aqueous fraction of the OraGRAFT^®^ product contains MMP2 activity together with the findings observed with a protease inhibitor propose a mechanism for how the TGF-β that is usually bound to the extracellular matrix is released into the supernatant [31]. The underlying mechanism required further research. Other questions are related to identifying other growth factors released from the OraGRAFT^®^ products. The bioassay presented here, based on IL11, NOX4, and PRG4 expression, is designed to show TGF-β activity, thus not covering the other growth factors released from DBM. Moreover, IL11 and NOX4 in particular are critically involved in mediating downstream TGF-β effects in cardiovascular and liver fibrosis [16,17] as well as systemic sclerosis [18]. However, further investigation is required to determine if IL11 and NOX4 mediate the TGF-β activity during bone regeneration. There is at least support for IL11 together with BMP-2 to accelerate bone regeneration [41] and for NOX4 to modulate osteoblast BMP-2 activity [42]. Additionally, PRG4 supports endochondral bone formation [43]. Future research should determine the possible role of the three selected genes to mediate part of the TGF-β activity released from allografts.

Future research should identify the proteomic signature of the aqueous solution of OraGRAFT^®^ and an overall allograft similar to what we have shown recently with acid bone lysates [23] and collagen-based biomaterials used for guided bone regeneration and soft tissue augmentation [44]. We should further consider a whole genome screening to identify all target genes, apart from IL11, NOX4, and PRG4, being activated in fibroblasts, for instance by gene arrays or RNAseq [23,29,30]. In line with this research strategy is to identify the overall cellular responses of progenitors of bone-forming osteoblasts and bone-resorbing osteoclasts. By this approach, we could identify further growth factors released by OraGRAFT^®^ products and uncover the mechanism involved in bone regeneration and thus graft consolidation. Finally, the possible variation of TGF-β activity depending on the lot requires attention in future studies. Thus, the data presented here are a primer for future research to broaden existing knowledge towards the refining and controlling manufacturing processes and to study the impact of allograft-derived TGF-β in the process of graft consolidation.

## 4. Materials and Methods

### 4.1. Preparation of Allograft Supernatants and Lysates

OraGRAFT^®^ Demineralized Cortical Particulate (1.2 cm^3^; IDs: 1817033-3066; 1817033-3079; 1817003-3080; 1817033-3086; and 1817033-3094) and OraGRAFT^®^ Prime consisting of moldable fibers of DBM (1.0 cm^3^; IDs: 1910583-3057; 1910583-3060; 1910583-3064; 1910583-3067; and 1910583-3069; LifeNet Health Europe GmbH, Vienna, Austria) were submerged in 4.8 mL and 8.0 mL, respectively, of serum-free Dulbecco’s Modified Eagle Medium (DMEM) for 72 h (Sigma Aldrich, St. Louis, MO, USA) and left under continuous shaking overnight at room temperature. Supernatants of each respective OraGRAFT^®^ were obtained upon centrifugation at 21,000 g for 10 min (Centrifuge 5420, Eppendorf, Hamburg, Germany). The supernatant of OraGRAFT^®^ Demineralized Cortical Particulate (SC) and OraGRAFT^®^ Prime consisting of moldable fibers (SF) were collected. The pelleted remaining allografts were resuspended in 1N HCl and left shaking for 72 h at room temperature. Acid lysates from each respective OraGRAFT^®^ (LC and LF) were collected through one centrifugation step. Their pH was then neutralized. All samples were stored in aliquots at –20 °C.

### 4.2. Cell Culture

Tissue samples of human gingiva were harvested from the extracted third molars of patients who had given informed and written consent. Prior to sample attainment, the Ethics Committee of the Medical University of Vienna (EK NR 631/2007) approved the protocol. In total, three strains of fibroblasts were established through explant cultures and less than 10 passages were used for the experiments. Cells were cultured in a humidified atmosphere at 37 °C in a growth medium consisting of DMEM, 10% fetal calf serum (Bio&Sell GmbH, Nuremberg, Germany), 1% of 10,000 units penicillin, and 10 mg of streptomycin/mL (Sigma Aldrich, St. Louis, MO). Cells were seeded at a concentration of 30,000 cells/cm^2^ onto culture dishes one day prior to stimulation. The stimulation was done by cell exposure to the supernatants and the lysates of the allografts. To examine the influence of TGF-β signaling, the inhibitor of TGF-β receptor type I kinase SB431542 (Calbiochem, Merck, Billerica, MA, USA) was used at 10 µM. Gene expression analysis and immunostainings were done as indicated. To identify the influence of proteases, the protease inhibitor cocktail tablet (Roche Diagnostics, Mannheim, Germany) was solved in 50 mL of serum-free DMEM and used for preparing the supernatant of OraGRAFT^®^ Prime. Gene expression analysis was done as indicated.

### 4.3. Cell Viability Assay

For the viability assay, gingival fibroblasts were seeded in the 96-well plate, then the following day, they were treated with 6 to 50% of aqueous solutions of the supernatants and acid lysates from both allografts overnight. For cell viability, MTT reagent (3-(4,5-dimethylthiazol-2-yl)-2,5-diphenyltetrazolium bromide; Sigma Aldrich, St. Louis, MO, USA) at a final concentration of 0.5 mg/mL was added to each well of a microtiter plate and incubated for 2 h at 37 °C, 5% CO_2_, and 95% humidity. The medium was removed, and the formazan crystals were solubilized with dimethyl sulphoxide (Sigma Aldrich, St. Louis, MO, USA). The optical density was measured at 570 nm. The data from independent experiments are presented as percentages of the optical density in the treatment groups normalized to the unstimulated controls that were considered 100% viability regardless of the optical density.

### 4.4. RT-PCR and Immunoassay

Total RNA was isolated with the ExtractMe total RNA kit (Blirt S.A., Gdańsk, Poland). Reverse transcription (RT) was performed with the LabQ FirstStrand cDNA Synthesis Kit (LabQ, Labconsulting, Vienna, Austria). Reverse transcription-polymerase chain reaction (RT-PCR) was done (LabQ, Labconsulting, Vienna, Austria) on a CFX Connect™ Real-Time PCR Detection System (Bio-Rad Laboratories, Hercules, CA, USA). Primer sequences are in Table 2. IL11 primer qHsaCEP0049951 was from Bio-Rad Laboratories, Inc. (Hercules, CA, USA). The mRNA levels were calculated by normalizing to the housekeeping genes GAPDH and 18s using the ΔΔCt method. The immunoassay was done with the TGF-β1 and human IL11 Quantikine ELISA kit (R&D Systems, Minneapolis, MN, USA).

### 4.5. Immunofluorescence

Gingival fibroblasts were plated in growth medium onto Millicell^®^ EZ slides (Merck KGaA, Darmstadt, Germany). The following day, cells were treated with serum-free medium overnight. The next day, cells were exposed to 30% of supernatants (SC and SF) with and without SB431542 for 30 min. Cells were then fixed in paraformaldehyde and blocked in 5% bovine serum albumin (BSA) and 0.3% Triton X-100 in phosphate-buffered saline (PBS) at room temperature, after which permeabilization with 0.1% Triton X-100 took place. Cells were incubated with Smad2/3 antibody (D7G7 XP^®^ rabbit mAb #8685, Cell Signaling, MA) overnight at 4 °C. Then, an Alexa Fluor^®^ 488-conjugated secondary antibody (Anti-Rabbit, Cell signaling Technology, Danvers, MA, USA) for 1 h at room temperature was used. Finally, cells were washed and mounted onto glass slides. Images were captured under a fluorescent microscope (Axio Imager M2, Carl Zeiss AG, Oberkochen, Germany).

### 4.6. Gelatin Zymography

Aqueous solutions of the supernatants and acid lysates from both allografts were mixed with Laemmli buffer and run on 10% sodium dodecyl sulfate (SDS-PAGE) gels containing 0.1% gelatin (Sigma Aldrich, St. Louis, MO, USA). After electrophoresis, gels were washed (50 mmol/L Tris HCl pH 7.5, 2.5% Triton X-100, 1 µM ZnCl_2_, 5 mmol/L CaCl_2_) two times for 30 min. Gels were then incubated overnight in incubation buffer (50 mmol/L Tris HCl pH 7.5, 1% Triton X-100, 1 μM ZnCl_2_, 5 mmol/L CaCl_2_) at 37 °C. The following day, gels were stained in Brilliant Blue R staining solution (Sigma Aldrich, St. Louis, MO, USA) for 30 min following destaining (methanol:acetic acid:water; 50:10:40). MMP2 activity appears as a clearance zone of digested gelatin within the stained gel at 72 kDa.

### 4.7. Statistical Analysis

Data points represent independent experiments with the median. The paired *t*-test was used to compare the two groups (SC and LC; SF and LF) in Figure 1, Figure 2, Figure 4 and Figure 7. For the data in Figure 3, we used the uncorrected Friedmann test against untreated controls. Analyses were performed using Prism v8 (GraphPad Software, La Jolla, CA, USA). *p*-Values are indicated in the figures.

## Figures and Tables

**Table 1:** The viability of fibroblasts exposed to the supernatants and acid lysates of allografts.

Concentration	50%	25%	12%	6%
SC	101.4 ± 0.2	97.8 ± 0.1	93.7 ± 0.1	86.5 ± 0.1
SF	85.7 ± 0.1	100.1 ± 0.3	121.7 ± 0.3	85.2 ± 0.1
LC	17.3 ± 0.04	13.5 ± 0.04	23.3 ± 0.01	104.0 ± 0.1
LF	20.0 ± 0.1	37.5 ± 0.2	111.5 ± 0.2	115.2 ± 0.2

Gingival fibroblasts were exposed to aqueous supernatants of OraGRAFT^®^ Demineralized Cortical Particulate (SC) and OraGRAFT^®^ Prime (SF), and the acid lysates of the two respective allografts (LC and LF). The data from the four experiments represent the percentage of remaining cell viability compared to an untreated control.

**Table 2:** The primer sequences

Primers	Sequence_F	Sequence_R
hNOX4	TCTTGGCTTACCTCCGAGGA	CTCCTGGTTCTCCTGCTTGG
hPRG4	CAGTTGCAGGTGGCATCTC	TCGTGATTCAGCAAGTTTCATC
hGAPDH	AAGCCACATCGCTCAGACAC	GCCCAATACGACCAAATCC
h18s	CCGATTGGATGGTTTAGTGAG	AGTTCGACCGTCTTCTCAGC

## Data Availability

All data are made available on demand.
